# Similar matrix alterations in alveolar and small airway walls of COPD patients

**DOI:** 10.1186/1471-2466-14-90

**Published:** 2014-05-26

**Authors:** Irene MJ Eurlings, Mieke A Dentener, Jack PM Cleutjens, Carine J Peutz, Gernot GU Rohde, Emiel FM Wouters, Niki L Reynaert

**Affiliations:** 1 Nutrition and Toxicology Research Institute Maastricht (NUTRIM), Department of Respiratory Medicine, University Hospital Maastricht, P.O. Box 5800, 6202 AZ Maastricht, The Netherlands; 2 Cardiovascular Research Institute Maastricht (CARIM), Department of Pathology, Maastricht University Medical Centre, Maastricht, the Netherlands

## Abstract

**Background:**

Remodelling in COPD has at least two dimensions: small airway wall thickening and destruction of alveolar walls. Recent studies indicate that there is some similarity between alveolar and small airway wall matrix remodelling. The aim of this study was to characterise and assess similarities in alveolar and small airway wall matrix remodelling, and TGF-β signalling in COPD patients of different GOLD stages.

**Methods:**

Lung tissue sections of 14 smoking controls, 16 GOLD II and 19 GOLD IV patients were included and stained for elastin and collagens as well as hyaluronan, a glycosaminoglycan matrix component and pSMAD2.

**Results:**

Elastin was significantly decreased in COPD patients not only in alveolar, but also in small airway walls. Interestingly, both collagen and hyaluronan were increased in alveolar as well as small airway walls. The matrix changes were highly comparable between GOLD stages, with collagen content in the alveolar wall increasing further in GOLD IV. A calculated remodelling index, defined as elastin divided over collagen and hyaluronan, was decreased significantly in GOLD II and further lowered in GOLD IV patients, suggesting that matrix component alterations are involved in progressive airflow limitation. Interestingly, there was a positive correlation present between the alveolar and small airway wall stainings of the matrix components, as well as for pSMAD2. No differences in pSMAD2 staining between controls and COPD patients were found.

**Conclusions:**

In conclusion, remodelling in the alveolar and small airway wall in COPD is markedly similar and already present in moderate COPD. Notably, alveolar collagen and a remodelling index relate to lung function.

## Background

Chronic obstructive pulmonary disease (COPD), currently the fourth leading cause of death, is characterised by reduction in expiratory airflow that is not completely reversible [1]. The major risk factor of COPD is cigarette smoking. Lung remodelling in COPD is marked by several characteristics, like accumulation of mucous secretions and bronchiolar fibrosis in the proximal airways and remodelling of small airway and alveolar walls. Small airways are most important in the reduction of FEV_1_. Importantly, remodelling of small airways is mainly associated with excess matrix deposition, whereas the neighbouring alveoli are hallmarked by destruction. Given these opposing remodelling processes, small airways and alveoli are mainly studied separately.

Small airway remodelling affects the transition zones between airway and alveolar spaces with both air-conducting and gas-exchange functions. Small airways are usually defined as < 2 mm in internal diameter without cartilage. They have the characteristically folded respiratory epithelium surrounded by a smooth muscle layer and supported by connective tissue without glands. Their thin walls offer little resistance to laminar airflow in healthy subjects [2]. Small airways obstruction in COPD is associated with airway wall thickening by remodelling related to tissue repair and accumulation of inflammatory exudates [3]. Evidence suggests that small airway remodelling arises as a result of epithelial abnormalities or from smooth muscle hypertrophy/hyperplasia [4-6]. Most studies show airway wall thickening based on image analysis. Studies into the molecular changes in extracellular matrix associated with small airway remodelling in COPD and mechanisms involved are few, and mainly focus on collagen, fibronectin and glycosaminoglycan deposition [7,8]. Also, thickening of the subepithelial layer of the airway wall, by increased expression of collagen I and III precursors was shown in airway wall biopsies of COPD patients, however very severe patients showed decreased precursor levels and a changed collagen I to collagen III ratio [9,10].

The main lesion in alveolar wall remodelling is emphysema, defined as “a condition of the lung characterised by abnormal, permanent enlargement of the airspaces distal to terminal bronchioles, accompanied by destruction of their walls and without obvious fibrosis” [11]. An imbalance between proteinases and their inhibitors is thought to account for the morphological changes [12].

Recently, studies have implicated that there might be more similarity between small airway and alveolar remodelling as previously thought. For instance, elastic fibres not only decreased in the alveolar but also in small airway walls in COPD [13]. Comparable extracellular matrix composition [14] and decreased levels of αSMA positive cells [15] in were found in parenchyma, small and large airways of mild to moderate COPD patients. Furthermore, fragmentation of the reticular basement membrane in COPD was shown as an increased number of clefts in small airways [16]. In addition, the number of small airways was decreased in patients with COPD [17], which indicates emphysema-like destruction of the small airways.

On the other hand some studies suggest that alveolar septae show signs of fibrosis, by accumulation or increased production of collagen [18] and proteoglycans [19,20]. The glycosaminoglycan hyaluronan, involved in maintaining the assembly of collagen fibrils and water homeostasis plays an important role in COPD. We previously demonstrated higher levels of hyaluronan, in both alveolar and airway walls of cigarette smoke exposed mice [21], and in sputum of COPD patients [22]. So far, none of these previously mentioned studies examined matrix remodelling simultaneously in different compartments of the lung and in both moderate and severe COPD patients.

TGF-β is an important regulator of extracellular matrix deposition, as it controls expression of components of the extracellular matrix network and of protease inhibitors. These combined anabolic and anti-catabolic effects make it a key growth factor in the development of tissue fibrosis. The main stimulatory signalling pathway involves association of pSmad2 and pSmad3, which in concert with Smad4 leads to activation of gene transcription of some main extracellular matrix components including collagens.

To characterise matrix remodelling and assess the role of TGF-β signalling in the distal lung, we examined small airway and remaining alveolar wall extracellular matrix content, phosphorylation of SMAD2 and their interrelation within subjects. To this end, area fractions of the major lung extracellular matrix components elastin, collagen and hyaluronan, and pSMAD2 were examined in small airway and alveolar walls. Furthermore the association between remodelling and lung function was assessed.

## Methods

### Study subjects and tissue collection

Lung tissue was obtained from the upper lobe subpleural area of 19 GOLD IV patients who underwent lung volume reduction surgery. From both 14 control subjects and 16 GOLD II patients, who underwent resection for a solitary peripheral tumour, tumour-free lung tissue in the subpleural area at appropriate distance from the tumour was taken. All lung tissue was obtained at University hospital Maastricht, the Netherlands, and tissue with a cross-sectional surface of approximately 2 cm^2^ was collected. Signs of a respiratory tract infection during 4 weeks preceding the study and a history of respiratory diseases, other than lung cancer were considered exclusion criteria. The number of pack years and the smoking status were recorded. People who stopped smoking at least 1 year prior to recruitment was considered an ex- smoker. All subjects had smoked at least 10 pack-years.

Lung function was determined by spirometry, and post-bronchodilator forced expiratory volume in 1 sec (FEV_1_) and forced vital capacity (FVC) were calculated from the flow-volume curve, and FEV_1_/FVC was calculated. Patients with FEV_1_/FVC < 0.70 and FEV_1_ between 50 and 80% were considered GOLD II, having FEV_1_ lower than 30% of normal were considered GOLD IV. All patients were prescribed combination therapy of inhaled corticosteroids and long-acting β2-agonists, tiotropium/ipratropiumbromide and salbutamol, on demand.

This study was conducted in compliance with the Helsinki Declaration. Lung tissue was obtained from the Maastricht Pathology Tissue Collection (MPTC). Collection, storage and use of tissue and patient data were performed in agreement with the “Code for Proper Secondary Use of Human Tissue in the Netherlands”. The scientific board of the MPTC approved the use of materials for this study under MPTC 2009–22.

### Staining

Four μm paraffin sections were cut from formalin fixed and paraffin embedded tissue.

For elastin staining, slides were incubated for 20 minutes in Weigert’s resorcin-fuchsin (Chroma, Muenster, Germany) at 60–70°C and counterstained in a 0.25% tartrazine (Chroma) in 0.25% acetic acid solution.

Collagen was stained by incubation for 90 minutes in 0.1% Picro Sirius Red, known to stain collagen I as well as II and III, in saturated aqueous picric acid, pH = 1.5 (Klinipath, Duiven, the Netherlands). Thereafter, sections were counterstained with haematoxylin.

For histolocalization of hyaluronan 2 μg/ml biotin-labelled hyaluronan binding protein was used (Calbiochem, Darmstadt, Germany) for 1 h. VECTASTAIN ABComplex/AP system (Vector, Burlingame, CA) was used for enzymatic reactivity and visualized with Vector Blue alkaline phosphatase substrate kit (Vector). Sections were counterstained with Nuclear fast red (Vector).

Phosphorylated SMAD2 was detected using a rabbit monoclonal Ab against human pSMAD2 (#3108, Cell Signalling Technology). After application of biotin-conjugated swine anti-rabbit IgG Ab (E-0431, DakoCytomation, Glostrup, Denmark) and alkaline phosphatase-labeled avidin–biotin complex (Vector), enzymatic reactivity was visualized using the Vector Blue Substrate Kit (Vector). Sections were counterstained with Nuclear Fast Red (Vector), mounted and pSMAD2 was semi-quantitatively scored by two blinded independent observers. Repeatability of the scoring was tested using the kappa coefficient which resulted in a value of κ = 0.79 which means a substantial agreement.

### Quantification of matrix

Sections were scanned using a .slide light microscopy slide scanner at 100 × magnification (Olympus, Hamburg, Germany) and analysed entirely using Leica QWin Pro version3.5.1 software (Leica Microsystems, Cambridge, United Kingdom) by a blinded observer. Per patient, 1 section of approximately 2 cm^2^ tissue, was divided in approximately 70 pictures, which were all analysed.

Alveolar staining was determined as percentage of stained area to total alveolar tissue area present on the slide. Therefore, first the total amount of tissue stained was detected followed by exclusion of blood vessels and airways. Thereafter, stained areas were selected and percentage of staining in the alveolar tissue was calculated by the software. Suitable small airways, defined as smaller than 2 mm diameter and cut in cross-sections by a ratio of maximal to minimal internal diameter < 2.0, were selected. From 14 controls, 13 GOLD II and 16 GOLD IV patients 2–4 airways per subject with a total of 111 airways were measured for which the amount was not significantly different between the study groups. Staining was calculated as percentage of stained area to total airway wall area. Airway wall thickness was measured from the lumen to the outer margin of the adventitia and calculated as mean wall width by the software. Staining intensity was validated using a pilot which contained five patients per group. All stainings were checked by a pathologist, who confirmed the quantification results as generated by the researcher as well.

An elastin to collagen-hyaluronan index was calculated to provide an estimation of ECM remodelling in terms of elastin, collagen and hyaluronan in COPD. This index, which was previously used in the lung [23], was adapted and defined as: [Elastin-(Collagen + Hyaluronan)]/[Elastin + (Collagen + Hyaluronan)] [23,24].

### Statistics

Results are displayed as mean ± SD. All data were normally distributed. Basic characteristics were analysed using ANOVA or Chi Square. Stainings were analysed using ANCOVA using age, sex, pack years and smoking status as covariates. Bonferoni correction was used for post-hoc analysis. Correlations were analysed by Pearson correlations (SPSS version 19.0, SPSS Inc., Chicago, IL). A p-value < 0.05 was considered significant.

## Results

### Subject characteristics

The clinical characteristics of the study subjects are summarized in Table 1. Controls and COPD patients were matched for age, but the ratio of current to ex-smokers differed significantly. Small airway wall width tended to be increased in GOLD II (23.9 ± 6.8) and GOLD IV (23.9 ± 9.9) compared to controls (19.8 ± 6.5); however this was not significant (p = 0.12).

**Table 1:** Clinical characteristics of study subjects

	**Control**	**GOLD II**	**GOLD IV**
**Number**	14	16	19
**Age (years)**	63 ± 6	65 ± 7	61 ± 7
**Sex (M/F)**	9/5	13/3 *$	12/7
**Pack-years §**	25 ± 16	46 ± 14*	47 ± 29*
**Smoking status (current/ex)**	4/9	11/5	0/19*^
**FEV**_**1 **_**(%predicted)**	107 ± 15	67 ± 9*	22 ± 4*^
**FEV**_**1**_**/FVC**	80 ± 6	59 ± 9*	26 ± 7*^
**Tlco (% predicted) #**	95 ± 16	77 ± 18*	35 ± 15*^

Data are displayed as mean ± SD or ratio, * means p < 0.05 compared to controls, $ means significantly different compared GOLD IV. ^ means significant compared to GOLD II. § Values from 3 controls, 4 COPD GOLD II and 2 GOLD IV patients are missing. # Values from 2 controls, 1 COPD GOLD II and 9 GOLD IV patients are missing. FEV_1_ (%predicted) and FEV_1_/FVC are measured post-broncholdilation.

### Decreased elastin in alveolar and small airway walls of COPD patients

**Figure 1:**
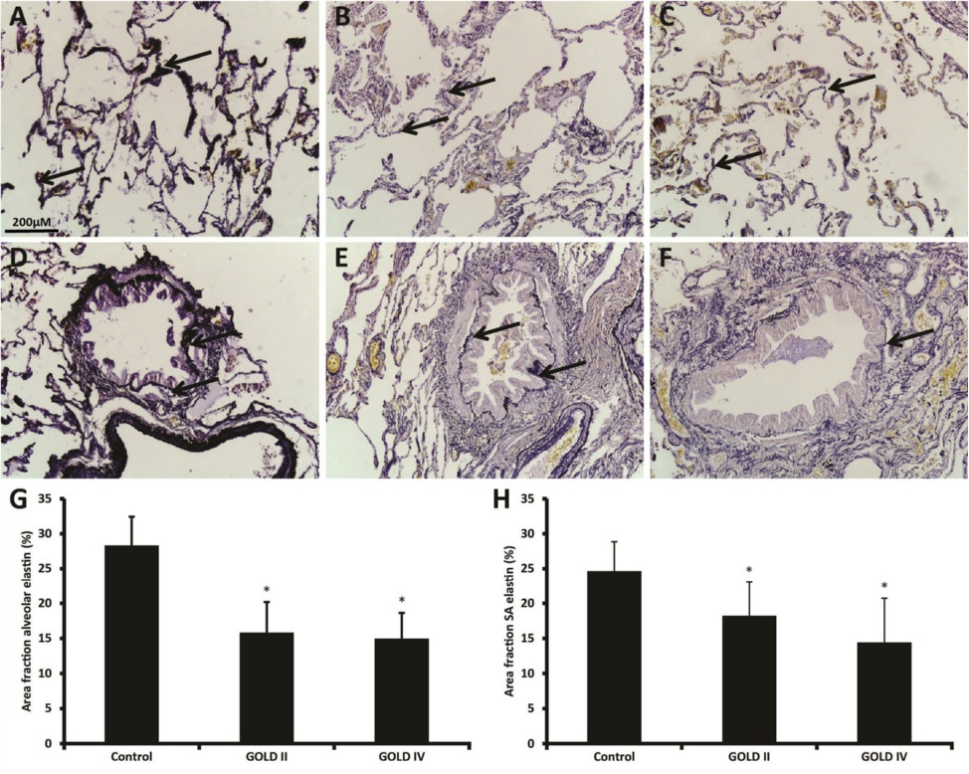
**Photomicrographs of elastin in alveolar walls of one representative control subject.** Photomicrographs of elastin in alveolar walls of one representative control subject **(A)**, GOLD II patient **(B)**, GOLD IV patient **(C)** and small airway walls of a control **(D)**, a GOLD II patient **(E)** and a GOLD IV patient **(F)**. Quantitative measured elastin content in alveolar walls **(G)** and small airway walls **(H)**. Data were analysed using ANCOVA and results are expressed as means + SD; *P < 0.05 compared to control.

Elastin is visible as dark purple strands (indicated by arrows) and is notable in alveolar walls, small airway walls around the epithelium and around blood vessels. We examined the amount of elastin in alveolar (Figure 1A-C) and small airway walls (Figure 1D-F) quantitatively (Figure 1G-H). As expected, the percentage of elastin in the alveolar walls was significantly reduced in GOLD II and GOLD IV. Interestingly, elastin was also decreased in the small airway walls of both GOLD II and GOLD IV. However, no difference was observed between GOLD II and GOLD IV patients for alveolar or small airway walls. There was a significant correlation between alveolar and small airway wall elastin values (Table 2).

**Table 2:** Correlations between alveolar matrix and small airway matrix in the whole study group

	**R**	**p**
**Elastin**	0.558	< 0.001
**Collagen**	0.612	< 0.001
**Hyaluronan**	0.523	< 0.001
**Remodelling index**	0.830	< 0.001
**pSMAD2**	0.656	< 0.001

Data were analysed using Pearson correlations.

### Deposition of collagen in the alveolar walls in addition to small airway walls

**Figure 2:**
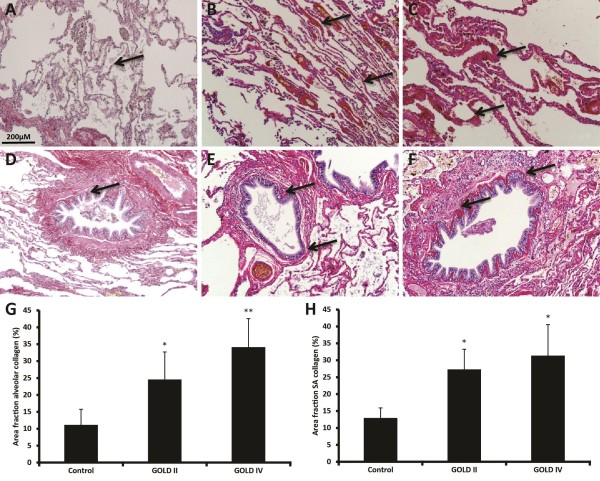
**Deposition of collagen in the alveolar walls in addition to small airway walls.** Photomicrographs of collagen in alveolar walls of one representative control subject **(A),** GOLD II patient **(B),** GOLD IV patient **(C)** and small airway walls of a control **(D),** a GOLD II patient **(E)** and a GOLD IV patient **(F)**. Quantitative measured collagen content in alveolar walls **(G)** and small airway walls **(H)**. Data were analysed using ANCOVA and results are expressed as means + SD; *P < 0.05 compared to control, **P < 0.05 compared to control and GOLD II.

Deposition of collagens (which could be either collagen I, II and/or III) indicated by arrows was observed in the pleura, perivascular, peribronchiolar in the basal membrane and connective tissue and in the alveolar walls (Figure 2A-F). Interestingly, in the alveolar wall collagen increased in GOLD II patients, which further progressed in GOLD IV (Figure 2G). As expected, it is shown that there is excess collagen deposition in the small airway walls of GOLD II and GOLD IV patients compared to controls; however no significant difference between GOLD stages was found (Figure 2H). In addition a significant correlation between alveolar and small airway collagen deposition was observed (Table 2).

### Increased hyaluronan in both alveolar and small airway walls of COPD patients

**Figure 3:**
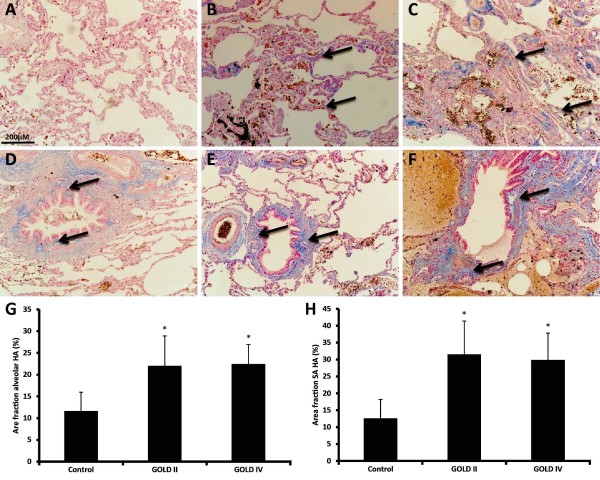
**Increased hyaluronan in both alveolar and small airway walls of COPD patients.** Photomicrographs of hyaluronan in alveolar walls of one representative control subject **(A)**, GOLD II patient **(B)**, GOLD IV patient **(C)** and small airway walls of a control **(D)**, a GOLD II patient **(E)** and a GOLD IV patient **(F)**. Quantitative measured hyaluronan content in alveolar walls **(G)** and small airway walls **(H)**. Data were analysed using ANCOVA and results are expressed as means + SD; *P < 0.05 compared to control.

Histochemical localization of hyaluronan was seen in the alveolar walls affiliated to capillaries; more prominent staining was observed in small airway walls and blood vessels (indicated by arrows), which was highly comparable to the localization of collagen (Figure 3A-F). Quantitatively enhanced hyaluronan was shown in alveolar (Figure 3G) and small airway walls (Figure 3H) of GOLD II and GOLD IV patients. No differences were found between GOLD II and GOLD IV patients. Again, a significant correlation between alveolar and small airway hyaluronan deposition was observed (Table 2).

### Elastin to collagen-hyaluronan index is decreased in COPD patients and correlates with lung function parameters

Next the relationship between individual matrix compounds and FEV_1_ was shown to be significant (Table 3). Correlations were present for all matrix components in alveolar and small airway walls with FEV_1_. However, in the COPD patients group alone, no significant correlations were found with exception of the inverse relation between alveolar collagen and FEV_1_.

**Table 3:** **Correlations between matrix and FEV**_**1**_

	**Whole study group n = 49**		**COPD patients n = 35**	
	**R**	**p**	**R**	**P**
**Elastin alveolar**	0.734	< 0.001	0.107	0.553
**Elastin small airway**	0.599	< 0.001	0.318	0.106
**Collagen alveolar**	−0.757	< 0.001	−0.511	0.003
**Collagen small airway**	−0.694	< 0.001	−0.219	0.262
**Hyaluronan alveolar**	−0.656	< 0.001	−0.232	0.201
**Hyaluronan small airway**	−0.434	< 0.001	−0.194	0.333

Data were analysed using Pearson correlations.

**Figure 4:**
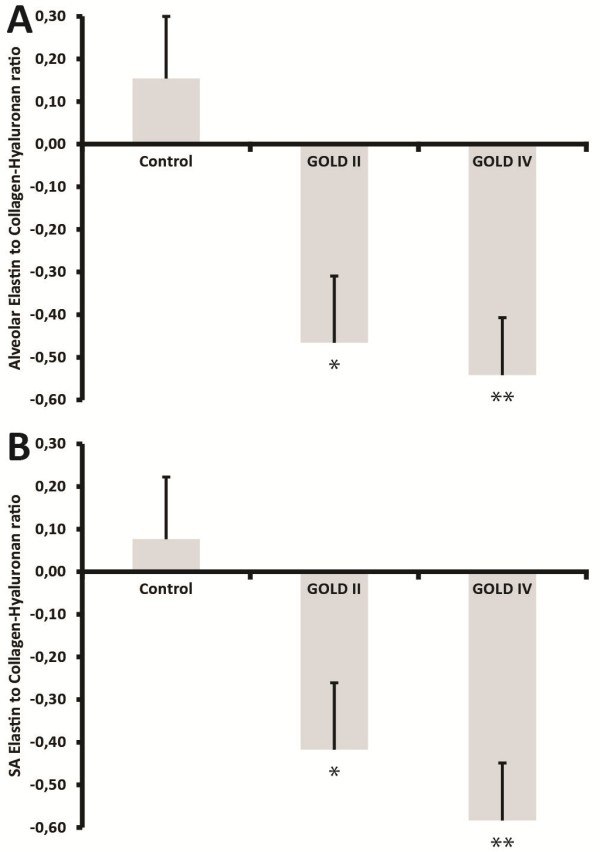
**Elastin to collagen-hyaluronan index is decreased in COPD patients.** Elastin to Collagen-Hyaluronan index of controls, GOLD II and GOLD IV patients in alveolar walls **(A)** and small airway walls **(B)**. Data were analysed using ANCOVA and results are expressed as means + SD; *P < 0.05 compared to control, **P < 0.05 compared to control and GOLD II.

**Figure 5:**
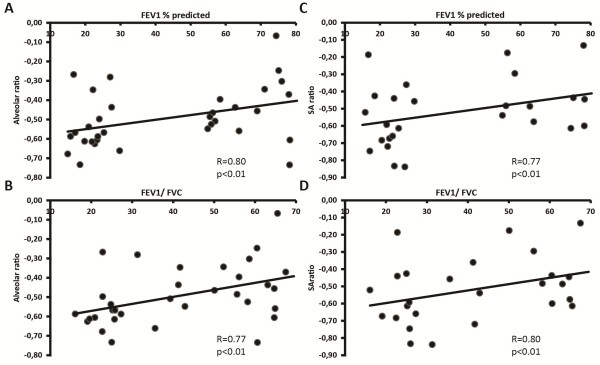
**Elastin to collagen-hyaluronan index correlates with lung function parameters.** Correlation in GOLD II and GOLD IV patients between alveolar elastin to collagen-hyaluronan index and FEV_1_**(A)**, FEV_1_/FVC **(B)** and between small airway elastin to collagen-hyaluronan index and FEV_1_**(C)**, FEV_1_/FVC **(D)**. Data were analysed using Pearson correlation.

An elastin-to-collagen-hyaluronan index was calculated to provide an estimation of the extent of ECM remodelling in COPD. Figure 4 shows that the index is lower in both GOLD II and GOLD IV patients than in controls. Furthermore, this index in contrast to the individual matrix components was significantly different between GOLD II and GOLD IV patients for both alveolar and small airway walls. Importantly, the remodelling index correlated with FEV1 as well as FEV1/FVC in COPD (Figure 5).

### Correlation between alveolar and small airway wall pSMAD2, despite absence of a relationship with COPD

**Figure 6:**
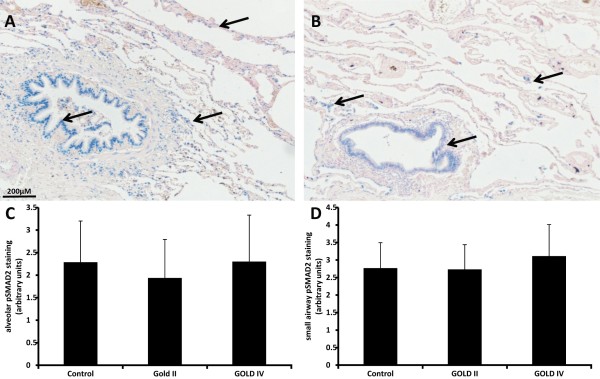
**Correlation between alveolar and small airway wall pSMAD2, despite absence of a relationship with COPD.** Photomicrographs of pSMAD2 staining in blue of one representative control subject **(A)** and a COPD patient **(B)**. Semi- quantitative scored pSMAD2 staining in alveolar walls **(C)** and small airway walls **(D)**. Data were analysed using ANCOVA and results are expressed as means + SD.

To assess if the described remodelling is associated with increased TGF-β signalling pSMAD2 was assessed by staining. Nuclear staining of pSMAD2 was seen throughout the lung, it was positive in alveolar macrophages and it was also observed associated with blood vessels, namely in the endothelial layer, in the smooth muscle cells of the media and in fibroblasts in the adventitia. For this study we focused on the staining which was observed in bronchial epithelium and its submucosa, as well as in fibroblasts, endothelial and type II epithelial cells in alveolar walls (Figure 6A-B). No difference was seen between controls and COPD patients in both small airway and alveolar walls (Figure 6C-D). Interestingly, a significant correlation between alveolar and small airway pSMAD2 staining was found (Table 2).

## Discussion and conclusion

This study was performed to compare matrix content in the distal lung of GOLD II patients, GOLD IV patients and controls. The data obtained show marked similarities in elastin, collagen and hyaluronan content changes between the alveolar and small airway compartment. Remarkably, only alveolar wall collagen was increased further in highly emphysematous patients compared to patients with moderate COPD and negatively correlated with lung function. All other matrix alterations were independent of lung function.

This study showed decreased elastin not only in alveolar but also in small airway walls of COPD patients. These results are in contrast with a study in severe GOLD IV patients showing increased elastic fiber density in alveolar walls [25]. This discrepancy could be caused by difference in control groups between these studies, which in our case were matched for age and smoking behaviour to the patient groups. In addition the data presented in this study showed a positive correlation of elastin content with lung function parameters using the whole study group. In COPD patients alone no significant correlations were determined. Previously, a study showed comparable correlations with lung function parameters, in the whole group including controls [13]. Combined with the lack of difference between GOLD II and GOLD IV patients, this attenuation of small airway elastin content is thus more likely a marker of disease, then of severity. A possible underlying mechanism for the decreased elastin content in the small airway wall is the increased number of neutrophils [26], especially neutrophil elastase [27]. In addition to loss of elastin, the number of small airways was reduced in mild and severe emphysematous patients [17]. The question remains whether the loss of elastin can lead to collapse or disappearance of small airways.

Deposition of collagen and hyaluronan was increased in small airway walls of COPD patients. This is an important finding as a number of studies showed small airway wall fibrosis, but there has been less research on the matrix composition. It was shown that there is an increase of collagen I and III precursors in the small airways of GOLD II patients; whereas in GOLD IV patients these precursors decreased [10], which can explain the lack of progression of collagen in the small airways as our data indicate. The current study shows elevated hyaluronan content in in addition to collagen. Within the lung collagen and elastin fibers are embedded in a hydrated gel of which glycosaminoglycans are the major constituents. The composition of the matrix and the fiber to gel ratio changes during maturation and disease state [28]. Hyaluronan is the major glycosaminoglycan in lung tissue and has diverse functions in lung homeostasis and pulmonary disease. We previously showed that hyaluronan was increased in sputum of COPD patients [22]. Furthermore in cigarette smoke exposed mice there was an increase in hyaluronan deposition in the small airway wall after 4 weeks of exposure, which also was not progressive in the 6 month exposure model of emphysema [21].

Increased collagen and hyaluronan deposition in the alveolar wall indicates that besides airway fibrosis there are fibrotic matrix changes in the alveolar walls which are also present in the remaining walls in severely obstructed patients with advanced emphysema. This is in line with previous research showing thickened alveolar walls in COPD patients and a decreased elastin-to-collagen index [23].

In this study two different disease phenotypes were analysed, a GOLD IV group with advanced emphysema and a moderately obstructed GOLD II group. We observed no difference between these groups for the individual matrix components, showing that changes in matrix composition even occur in patients with moderate airflow limitation. Only collagen in alveolar walls was further increased in severely emphysematous patients. In addition, collagen area fraction in the alveolar walls was the only measure which correlated with TLCO within the COPD group (R = −0.493, p = 0.02). This indicates that next to destruction of alveolar walls, increased collagen content in remaining walls contributes to reduction of gas transfer. Unfortunately, correction for the amount of alveolar tissue left in these patients was impossible. In an elastase model it was shown that decreased elastin caused increased alveolar collagen without inflammation. Furthermore, it appears that remodelled collagen fibres are weaker and break under the influence of mechanical stress [29]. The mechanism underlying remodelling of elastin and collagen, including subtyping composition and ultrastructure, should be investigated to support their involvement in emphysema development.

In addition, a significantly decreased remodelling index in COPD patients was found. This index was also significantly different for both small airway and alveolar walls of COPD patients versus controls, in line with individual matrix components. More interestingly there was a significant difference in the remodelling index between GOLD II and GOLD IV patients which positively correlated with FEV_1_ and FEV_1_/FVC in the patient group. Although the distribution was high, possibly due to the heterogeneous GOLD II population, the correlations were strong and significant. The individual matrix components on the other hand did not differ significantly between GOLD stages and did not correlate with lung function, indicating that although the individual components are markers of disease, even in early stages, it is the combination of matrix component alterations that is related to progressive airflow limitation.

The amount of pSMAD2 staining in alveolar and small airway walls was not different between controls and COPD patients. Literature on the role of TGF-β in COPD is conflicting; some studies found increased expression of TGF-β in bronchial and alveolar epithelial cells [30,31] whereas others observed no differences in TGF-β levels in sputum, BALF and bronchial epithelial cells by immunostaining [32-36]. The lack of difference in pSMAD2 staining is in line with these latter publications [35]. In the smoking mouse model of COPD there is evidence for increased TGF-β expression and SMAD signaling [37]. Furthermore increased levels in TGF-β expression were shown between never and current smokers [32] while no difference between smokers and COPD patients was present. In this study using smoking controls, no differences were found in relation to the disease. An effect of smoking per se cannot be excluded given the lack of a never smoking control group.

The observed correlation between alveolar and small airway wall matrix components invalidates previous thoughts that alveolar and small airway remodelling are two distinct processes which is in concert with some recent publications [13,16,17]. The finding that elastin is decreased in both alveolar and small airway walls and that collagen and hyaluronan are increased in both compartments provides evidences that there are similar matrix adaptations occurring simultaneously in alveoli and small airways. Furthermore, a positive correlation was found in pSMAD2 staining between alveolar and small airway walls, indicating that not only remodelling in the small airways and alveolar walls can be seen as one unit but that also TGF-β signalling as shown by pSMAD2 is similar. The current study indicates that remodelling and TGF-β signalling in the alveolar and small airway wall in COPD are markedly similar and that remodelling is already present in moderate COPD. Notably, alveolar collagen and a remodelling index relate to progressive airflow limitation.

## Competing interests

The authors declare that they have no competing interests.

## Authors’ contributions

IE performed immunohistochemical stainings, quantitative scoring and statistical analysis, and drafted the manuscript. MD supervised experiments, participated in semi-quantitative immunohistochemical analysis and drafted the manuscript. JC designed the programs for quantitative scorings. CP validated and assessed immunohistichemical stainings. GR participated in the design of the study and drafted the manuscript. EW participated in the design of the study, provided general supervision and drafted the manuscript. NR participated in the study design, interpretation of data and manuscript preparation. All authors read and approved the final manuscript.

## Pre-publication history

The pre-publication history for this paper can be accessed here:

http://www.biomedcentral.com/1471-2466/14/90/prepub
